# Symptom trajectories in patients with panic disorder in a primary care intervention: Results from a randomized controlled trial (PARADISE)

**DOI:** 10.1038/s41598-019-43487-x

**Published:** 2019-05-09

**Authors:** Karoline Lukaschek, Thomas S. Hiller, Ulrike Schumacher, Tobias Teismann, Jörg Breitbart, Christian Brettschneider, Hans-Helmut König, Jürgen Margraf, Jochen Gensichen

**Affiliations:** 1 Institute of General Practice and Family Medicine, University Hospital of Ludwig-Maximilians-University Munich, Pettenkoferstr. 10, D-80336 Munich, Germany; 2 Institute of General Practice and Family Medicine, Jena University Hospital, Bachstrasse 18, D-07743 Jena, Germany; 3 Centre for Clinical Studies, Jena University Hospital, Jena, Salvador-Allende-Platz 27, D-07747 Jena, Germany; 4 Mental Health Research and Treatment Center, Ruhr-Universität Bochum, Massenbergstr. 11, D-44787 Bochum, Germany; 5 Department of Health Economics and Health Services Research, Hamburg Center for Health Economics, University Medical Center Hamburg-Eppendorf, Martinistrasse 52, D-20246 Hamburg, Germany

**Keywords:** Outcomes research, Medical research

## Abstract

This analysis aims to identify and characterize symptom trajectories in primary care patients with panic disorder with/without agoraphobia (PD/AG) who participated in a primary care team based training involving elements of cognitive behavioural therapy (CBT). Growth Mixture Modeling was used to identify different latent classes of change in patients with PD/AG (N = 176) who underwent treatment including CBT elements. We identified three patient classes with distinct similar trajectories. Class 1 (n = 58, mean age: 46.2 years ± 13.4 years, 81% women) consisted of patients with an initially high symptom burden, but symptoms declined constantly over the intervention period. Symptoms of patients in class 2 (n = 89, mean age: 44.2 years ± 14.5 years, 67.4% women) declined rapidly at the beginning, then patients went into a plateau-phase. The third class (n = 29, mean age: 47.0 years ± 12.4 years, 65.5% women) was characterized by an unstable course and had the worse outcome. Our findings show that only a minority did not respond to the treatment. To identify this minority and refer to a specialist would help patients to get intensive care in time.

## Introduction

In primary care settings, up to 6.8% of patients are diagnosed with panic disorder (ICD-10: F41.0)^1^. Agoraphobia (ICD-10: F40.01) is comorbid to panic disorder in 35–65% of all cases^2^. Cognitive-behavioural therapy (CBT) can be effective for the treatment of panic disorder with or without additional agoraphobia (PD/AG) (e.g.^3,4^). General practitioners (GPs) can deliver key-elements of CBT to patients with PD/AG as a first step in treatment^5^, such as psychoeducation, coping skills, cognitive restructuring, and self-managed exposure exercises^6^.

Knowledge regarding the course of PD/AG could have major implications for treatment outcome. However, it is difficult for GPs to predict the course of a patient’s illness over time. Knowledge about differences in patterns of change in the trajectories for specific subgroups of patients might enable GPs to maximize treatment outcome in individual patients and to optimize the cooperation with mental health specialists^7,8^. Pattern recognition based on the similarities of the change trajectories shared by a group of patients can be identified via growth mixture modelling (GMM) (e.g.^9,10^). Once identified as being a member of a certain group, or class, patients cannot move between classes.

Very few studies have investigated treatment response patterns in the trajectories of patients suffering from PD/AG using GMM. Lutz *et al*. (2014) were among the first to analyse long-term strategies in the treatment of PD/AG^7^. In their study of patients (N = 326) undergoing CBT, four latent patient subgroups were identified, showing clusters of change trajectories over the first few treatment sessions. The subgroup with early positive change was likely to be reliably improved at the end of the treatment. Lutz *et al*. concluded that early treatment changes are uniquely predictive of treatment outcome^7^.

The present analysis was conducted within the framework of the Jena-PRARDIES study in which practice team-supported exercises with case management could better improve symptoms of PD/AG in primary care patients than treatment as usual^11^. As part of the case management, medical assistants (MAs) periodically monitored patients by telephone using a checklist to assess symptoms and encouraging treatment adherence after a brief CBT-intervention^12^.

The aims of the present analysis were to (1) examine associations between symptom trajectories and baseline characteristics, (2) identify and characterize treatment response patterns in the trajectories of patients suffering from PD/AG who participated in a primary care team based training involving elements of CBT.

## Methods

### Study design and participants

Data were derived from the “Patient Activation foR Anxiety DISordErs” (PARADISE) study^6^, a cluster randomized controlled trial comparing a practice team-supported, self-managed exposure program for patients with PD/AG in general practices to usual care [Trial Registration: Current Controlled trials ISRCTN64669297, 07/11/2012; Deutsches Register Klinischer Studien DRKS00004386, 25/09/2012]. Patients in the intervention group showed a significant reduction in anxiety symptoms compared to patients in the control group (*accepted for review*). The study was approved by the ethics committee of the Friedrich- Schiller-University at the Medical Faculty (Jena, Germany). The study was planned and conducted in consideration of Good Clinical Practice guidelines (ICH Topic E6, 2002) as well as in accordance with the medical professional codex and the Helsinki Declaration as updated in 2013. Informed consent was obtained from all participants. Details of the study design and recruiting process have been published^6^.

To be eligible for the trial, patients had to meet the following inclusion criteria: (1) being at least 18 years of age, (2) being diagnosed with PD/AG (ICD-10: F41.0 or F40.01), (3) showing a minimum total score on the ‘Overall Anxiety and Impairment Scale’ (OASIS) of 8 points^13^ and at least two positive answers on the panic module of the ‘Patient Health Questionnaire’ (PHQ^14^), (4) having sufficient German language skills, (5) having a private telephone, (5) being capable of giving written informed consent to participate in the study. Patients were excluded if they met one or more of the following exclusion criteria: suffering from acute suicidal tendencies, acute or chronic psychosis, dependence on psychoactive substance(s), or severe physical illness; being pregnant; receiving professional psychotherapeutic treatment for their anxiety disorder at the time of inclusion. We used primary care practices (clusters) as units of randomization and performed a 1:1 cluster randomization^6^.The randomization list was computer-generated and concealed from the study team. Patients were blinded to their practice’s allocation status until after baseline assessment.

In total, the trial included 419 patients suffering from PD/AG (mean [SD] age, 46.2 [14.4] years; 74% female). In the present analysis of the intervention group (36 practices, 230 patients) 176 patients (mean age: 45.3 years ± 13.8 years, 71.6% women) with data on telephone monitoring sessions and visits to the GP were included.

### Intervention

Practice-team supported exposure training involved evidence-based CBT elements (psychoeducation and CBT-oriented exposure exercises)^15^ and elements derived from the chronic care model^16^. Patients in the intervention group received a workbook containing psychoeducational information, instructions on conducting exercises, and exercise protocol forms. Over 23 weeks, GPs provided four structured sessions, the first three of which were designed to individualize the major CBT elements (psychoeducation, interoceptive exposure exercises, and situational exposure exercises)^15^. Starting from session two, patients were instructed to practice exposure exercises at least twice a week. At session four, patients were provided with relapse-prevention information. The MA periodically monitored the patients by telephone using a checklist^12^. Data from this checklist were used to identify patterns of change over the course of treatment.

### Outcome measure

The target variable for the present analysis was anxiety severity, measured by the short Overall Anxiety Severity and Impairment Scale (OASIS)^13^. The short OASIS is a 5-item measure that assesses frequency of anxiety, intensity of anxiety symptoms, behavioral avoidance, and functional impairment associated with anxiety. The original short version of the OASIS was validated in a in a large sample (N = 1,036) of primary care patients and showed good reliability and validity^17^.

Respondents select among five different response options for each item, which are coded 0 to 4 and summed to obtain a total score (range: 0–20). A cut-score of 8 correctly classifies 87% of a clinical population sample as having an anxiety diagnosis or not^13,18^. The German version of the OASIS used in this study was validated in a sample of primary care patient and showed good psychometric properties^19^.

### Statistical methods

Growth mixture modelling (GMM) is an advanced cluster analytic method that allows categorizing individuals into subgroups following similar change trajectories over a defined period. It isolates groups of patients with similar treatment response patterns or profiles over time^20^. We identified latent subgroups (“classes”) of treatment effect trajectories by fitting GMM with patient as random factor and time, i.e. baseline and 10 visits during ongoing treatment, as fixed factor. Models incorporating practices as clusters were also considered but did not improve model fit. Since regular telephone monitoring including assessment of OASIS was part of the intervention, only patients of the intervention-arm were included in the analysis. Categorical latent variables were used to identify classes of trajectories within the patient population. Two- to five-class models were analysed, the best model was chosen based on Akaike Information Criterion (AIC) and Bayesian Information Criterion (BIC) as well as by medical plausibility. We identified a two-class model with the best goodness of fit according BIC, the most restrictive criterion with respect to number of classes. According AIC, the three and four-class models show a better fit, however, the latter retains mainly the three classes, but additionally segregating a fourth class containing only 3 patients with very alternating values. Consequently, from medical point of view, the three-class model shows better interpretability.

Statistical analyses were conducted using SPSS 23 for data capturing, Mplus Version 8 for GMM and SAS 9.4 for other analyses.

### Trial registration

Current Controlled Trials [http://www.isrctn.com/ISRCTN64669297]; Deutsches Register Klinischer Studien [https://www.drks.de/drks_web/navigate.do?navigationId=trial.HTML&TRIAL_ID=DRKS00004386].

## Results

Our sample of primary care patients suffering from PD/AG was predominantly female (71.6%), middle aged (mean age: 45.3 ± 13.8 years), and well educated (mean years of education: 11 ± 2.9 years).

### Associations between symptom trajectories and baseline characteristics

We identified three classes of patients with distinct similar trajectories. The class-characteristics are shown in Table 1. Class 1 had a statistically significant higher proportion of women (p = 0.004, Fisher’s exact test). We observed no differences between classes regarding mean age and years of education. Patients of class 3 were more likely to live in an urban than in a rural area. (p = 0.02, Fishers exact test). Class 2 had statistically significant lower OASIS values at baseline (as shown by confidence interval for the mean). Regarding depression, there were statistically significant differences between classes (p < 0.001, Fisher’s exact test); in class 3, 35.7% of the patients suffered from major depressive disorder compared to 12.2% (class 1) and 2.5% (class 2).

**Table 1:** Patients’ characteristics of all classes of the three-class solution.

Parameter		Class 1 (n = 58)	Class 2 (n = 89)	Class 3 (n = 29)	Total (N = 176)
Sex	Female (n/%)	47/81.0	60/67.4	19/65.5	*
Age [years]	Mean	46.2	44.2	47	45.3
	StdDev	13.4	14.5	12.4	13.8
	95%-LClm	42.6	41.2	42.2	°
	95%-UClm	49.7	47.3	51.7	
Years of education	Mean	10.9	11	11.2	11
	StdDev	2.7	3	2.7	2.9
	95%-LClm	10.1	10.4	10.2	°
	95%-UClm	11.6	11.7	12.2	
OASIS (baseline)	Mean	12.4	8.7	11.2	10.4
	StdDev	2.8	3.8	3.8	3.9
	95%-LClm	11.7	7.9	9.8	**
	95%-UClm	13.2	9.5	12.7	
Depression (PHQ9)	Major depressive disorder (n/%)	6/12.2	2/2.5	10/35.7	***
	Other (n/%)	5/10.2	6/7.6	3/10.7	
Place of residence	Urban (n/%)	12/20.7	22/24.7	8/27.6	****
	Rural (n/%)	46/79.3	67/75.3	21/72.4	

95%-LClm = 95% Lower Confidence limit, 95%-UClm = 95% Upper Confidence limit, StdDev = Standard Deviation.

°no statistically relevant difference.

*Class 1 has a statistically significant higher fraction of women (p = 0.004, Fisher’s exact test).

**Class 2 has statistically significant lower OASIS values at baseline (as shown by confidence interval).

***Differences between classes with respect to depression at baseline are statistically significant (p < 0.001, Fisher’s exact test).

****Class 3 was more likely to live in an urban area (p = 0.02, Fisher’s exact test).

### Characteristics of symptom trajectories, three-class solution

**Figure 1:**
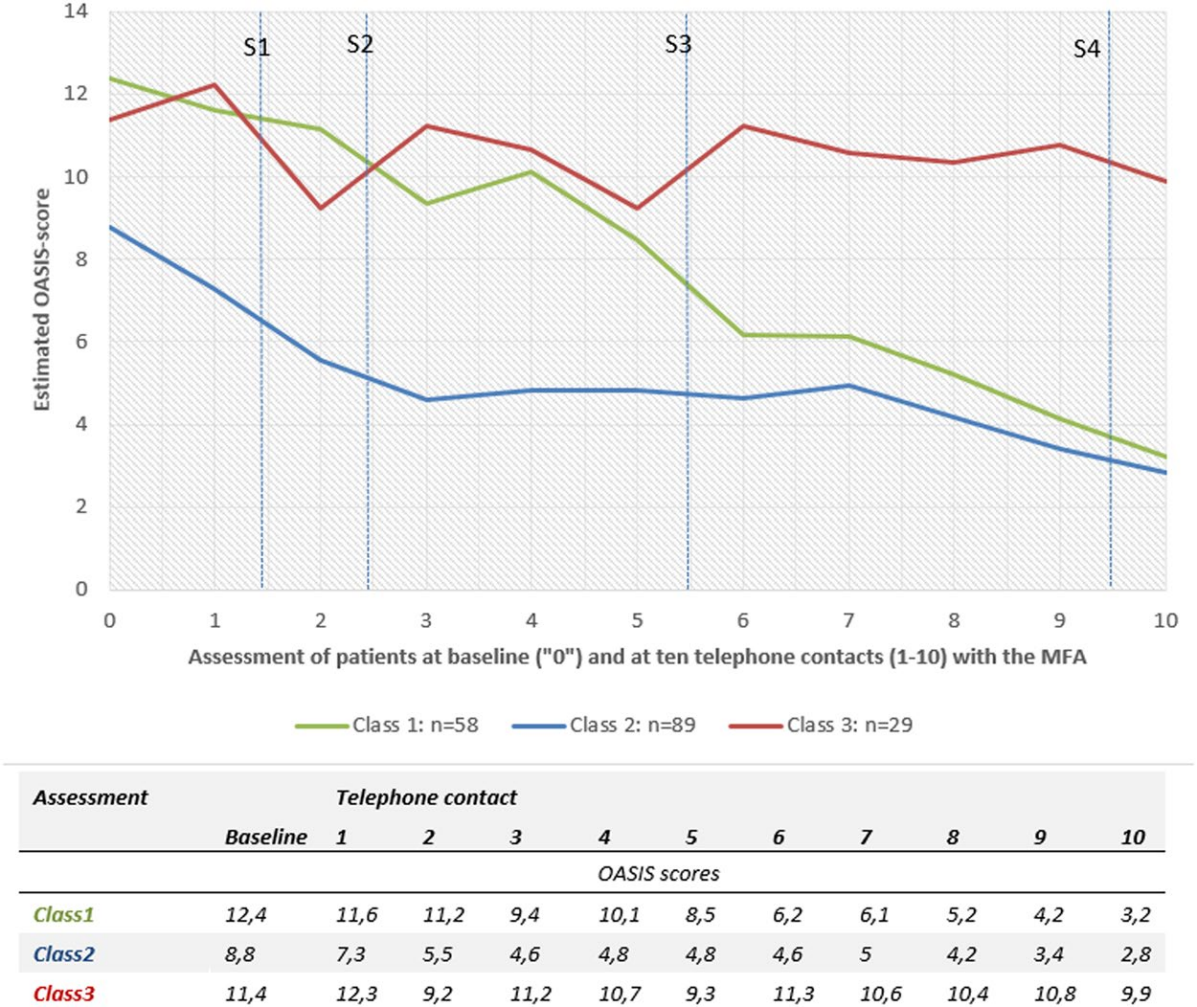
Growth mixture modelling (GMM), OASIS-scores estimated by the three-class model. Vertical lines indicate four sessions (S1–S4) with the GP (session 1, week 1: Psychoeducation; session 2, week 6: interoceptive exposure exercises; session 3, week 12: situative exposure exercises; session 4, week 20: relapse prophylaxis). X-axis indicates baseline assessment (“0”) and 10 telephone assessments by the MA (1–10). Y-axis indicates estimated OASIS-Score.

Figure 1 shows the estimated latent growth curves for the three-class solution including baseline assessment (T0) and 10 telephone assessments.

Class 1 (n = 58, mean age: 46.2 years ± 13.4 years, 81% women) had the highest estimated OASIS scores at baseline (12.4). The estimated score declined to 3.2 at telephone assessment 10. Class 1 showed improvement over almost all assessments, with a small peak at telephone assessment 4 (estimated score: 10.1).

Class 2 (n = 89, mean age: 44.2 years ± 14.5 years, 67.4% women) started with a lower estimated OASIS score about 8.8 and is marked by a steep decline to a score of 4.6 at telephone assessment 3. This is followed by a stable phase until telephone assessment 7, from which on the OASIS score gently drops to 2.8 (telephone assessment 10).

Class 3 (n = 29, mean age: 47.0 years ± 12.4 years, 65.5% women) starts at a rather high estimated OASIS score of 11.4 at baseline. In this class, a consistent pattern is missing, and its zigzag course seems rather unsteady. This class ends with a score of 9.9 at telephone assessment 10.

*Sessions with the GP* are indicated as vertical lines in Fig. 1. The OASIS scores declined in all classes after the first session (psychoeducation). Class 1 continued to improve throughout the course of the intervention. Class 2 showed further improvement after session 2 (start of interoceptive exposure exercises), but none directly after session 3 (start of situational exposure exercises). In contrast, class 3, the smallest and most unstable subgroup, showed an increase in anxiety severity after GP sessions 2 and 3.

Looking at the numbers of therapeutic contacts with the GP(Table 2), it shows that all class 3 patients (n = 29) attended sessions 1 and 2, and that only one class 3 patient missed session 3. Patients in class 1, on the other hand, were more likely to skip sessions and less likely to attend all four sessions with the GP. Class 2 has a higher proportion of patients who attended session 4 than classes 1 or 3. In general, session 4 had the highest percentage of non-attenders in all three classes.

**Table 2:** Number of therapeutic contacts by trajectory - ITT, intervention group, three-class model.

Question	Answer	Class 1		Class 2		Class 3		Total	
		N	%	N	%	N	%	N	%
Session 1 performed	No	2	3.4	.	.	.	.	2	1.1
	Yes	56	96.6	89	100.0	29	100.0	174	98.9
Session 2 performed	No	4	6.9	6	6.7	.	.	10	5.7
	Yes	54	93.1	83	93.3	29	100.0	166	94.3
Session 3 performed	No	12	20.7	12	13.5	1	3.4	25	14.2
	Yes	46	79.3	77	86.5	28	96.6	151	85.8
Session 4 performed	Missing	.	.	.	.	1	3.4	1	0.6
	No	17	29.3	23	25.8	7	24.1	47	26.7
	Yes	41	70.7	66	74.2	21	72.4	128	72.7

## Discussion

In our sample of predominantly female, middle-aged primary care patients with PD/AG who received four CBT-oriented GP sessions and 10 accompanying MA-telephone assessments, we identified three different trajectories: a class of patients with highest anxiety symptoms at treatment start, who responded slowly and seemed to benefit from the whole intervention programme as indicated by a continuous improvement of anxiety symptoms (class 1); a class of patients with lowest anxiety levels who responded early to treatment, then went into a plateau-phase and ended with a final improvement similar to class 1 (class 2); a third class that did not seem to benefit from the intervention programme as indicated by a fluctuating anxiety symptom burden and only minimal improvement over time (class 3).

A previous study^7^ using GMM to identify trajectories of anxiety found four patterns of early treatment response comparable to ours: a rapidly improving group, an initially highly symptomatic and slowly improving group, an initially low symptom and slowly improving group, and an early deteriorating group^7^. These response profiles were predictive of treatment outcome and, to a lesser degree, the number of sessions attended. In our study, class 1 was less likely to complete all four sessions with the GP, probably because these patients’ condition continuously improved. On the other hand, class 3 most reliably kept their sessions, probably due to help-seeking behaviour caused by these patients’ bad condition. Due to the high risk for treatment drop-out of such patients showing no improvement, only few other studies that investigated change patterns with a cluster analytic method reported this specific subgroup^20,21^. This subgroup is of clinical importance, because it clearly showed the worst treatment outcomes and still had high levels of symptom severity at the end of the intervention. The non-response to the treatment became obvious for the GP around session 3, but was in the looming after session 2 as indicated by the unsteady course. GPs should act quickly and refer these patients to a mental health specialist (“stepped care”); obviously, they cannot be helped with a low-threshold minimal treatment administered by GPs. This group was also burdened with the highest percentage of major depressive disorder at baseline. The combination of high anxiety levels and major depressive disorder might be a further indication for referral to a specialist.

Monitoring by the MA and sessions with the GP had a positive effect on class 1. Regarding class 2, GP sessions 3 and 4 apparently did not have an influence; we assume that monitoring by the MA prevented a deterioration of PD/AG. However, we cannot exclude that a more intense case management, e.g. by a psychotherapist, could have led to further improvement of class 2.

### Strengths and limitations

The strengths of this study are its embedding within the framework of the thoroughly designed Jena-PARADIES study and the use of a well-established anxiety instrument. In contrast to other models, e.g. the staging model^22^, using GMM to identify classes can provide information whether an intervention is effective, while stages provide information on the individual disease burden. A limitation of the present analysis relates to the interpretation of the early positive response pattern. It cannot be ruled out that early response was also partially due to factors such as regression to the mean or placebo effects^23^. To eliminate these alternative explanations, it would be necessary to investigate additional change patterns among an untreated group of patients with PD/AG, and compare the patients within early positive change classes. In the present analysis, this was not possible, because we do not have data on trajectories in the control group.

## Conclusion

Our findings show that for the majority of anxiety patients a low-threshold treatment administered by the GP is adequate. Only a minority did not respond to the treatment. To identify this minority and refer to a specialist would help patients to get intensive care in time.

## Supplementary information

CONSORT Checklist

## Data Availability

The authors confirm that, for approved reasons, access restrictions apply to the data underlying the findings and thus they cannot be made freely available in the manuscript, the Supplemental Files, or a public repository. The data are subject to national data protection laws and restrictions were imposed by the Ethics Committees to ensure data privacy of the study participants. However, they can be applied for through an individual project agreement with the PI of the study, Prof. Gensichen (Jochen.Gensichen@med.uni-muenchen.de).
